# Patient level barriers to accessing TB care services during the COVID-19 pandemic in Uganda, a mixed methods study

**DOI:** 10.1186/s12913-023-10513-8

**Published:** 2024-01-10

**Authors:** Mudarshiru Bbuye, Stella Zawedde Muyanja, Isaac Sekitoleko, Roma Padalkar, Nicole Robertson, Madeline Helwig, Dennis Hopkinson, Trishul Siddharthan, Peter Jackson

**Affiliations:** 1 Makerere University Lung Institute, College of Health Sciences, Makerere University, Kampala, Uganda; 2 Infectious Disease Institute, College of Health Sciences, Makerere University, Kampala, Uganda; 3 MRC/UVRI and LSHTM Uganda Research Unit, Entebbe, Uganda; 4 London School of Hygiene and Tropical Medicine, London, UK; 5 Hackensack University Medical Center, New Jersey, USA; 6 Department of Medicine, Johns Hopkins School of Medicine, Baltimore, MD USA; 7 Virginia Commonwealth University School of Medicine, Richmond, VA USA; 8 Division of Pulmonary and Critical Care, Department of Medicine, Virginia Commonwealth University, Richmond, USA; 9 Division of Pulmonary and Critical Care, Duke University, Durham, NC USA; 10 Division of Pulmonary and Critical Care, University of Miami, Miami, FL USA

**Keywords:** Tuberculosis, COVID-19 pandemic, Lockdown, Uganda, Qualitative, Quantitative, Mixed methods

## Abstract

**Introduction:**

Lockdown measure has been utilized widely to mitigate COVID-19 pandemic transmission and recently during the 2022 Sudan Ebola Virus Disease outbreak in Uganda. These have setback effects on the continuity of essential health services such as tuberculosis (TB) care, reversing progress made in the fight against tuberculosis (TB) over the past decade. We set out to understand patient-reported barriers to accessing TB care services during the COVID-19 pandemic in Uganda.

**Methods:**

Mixed methods study involving review of medical records of TB patients who received TB care from January to September 2020. We used quantitative and qualitative methods including phone questionnaires and in-depth interviews. We carried out descriptive statistics, a chi-square test and conducted a thematic analysis.

**Results:**

We carried out phone interviews with 672 participants. The majority (60%) were male and with an average of 35 years (SD:11). A significantly higher proportion of patients reported a barrier to TB care access during the COVID-19 lockdown than pre-lockdown (79.9% vs. 68.1% *p* = 0.027). We carried out in-depth interviews with 28 participants (54% (15/28): male). Barriers experienced by these participants included lack of a means of transport to reach the health facility, lack of money to pay the transport fares, long distances to the facility, fear of COVID-19 infection, stigma due to overlap between TB and COVID-19 symptoms, and few health care workers available during the lockdown period.

**Conclusion:**

Lockdown measures instituted to mitigate the transmission of COVID1-19 affected access to TB care services in Uganda. Uganda is at risk of future emerging and re-emerging diseases of epidemic potential. Therefore, there should be measures to ensure the continuity of essential services such as tuberculosis care during the implementation of future epidemic response interventions such as a lockdown.

**Supplementary Information:**

The online version contains supplementary material available at 10.1186/s12913-023-10513-8.

## Background

Lockdown measure has been utilized widely to mitigate COVID-19 pandemic transmission and recently during the 2022 Sudan Ebola Virus Disease outbreak in Uganda [1, 2]. These have setback effects on the continuity of essential health services such as tuberculosis (TB) care, reversing progress made in the fight against tuberculosis (TB) and other endemic diseases over the past decade. Globally, the number of people newly diagnosed with TB reported to the WHO fell by almost 20% from 7.1 million in 2019 to 5.8 million in 2020 and recovered to 6.4 million in 2021; these fluctuations have been primarily attributed to the COVID-19 pandemic [3]. While diagnoses fell, the number of deaths from the disease increased by 7% during the same period [4]. Much of the disruption to TB service delivery is a result of wider disruptions in social and economic systems brought about by restrictions in movement and closure of public services e.g. schools, workplaces, and public transport. This disruption is not limited to COVID-19 and future and current infectious disease outbreaks such as the recent 2022 Sudan Ebola outbreak in Uganda and elsewhere have the potential to disrupt well-established treatment programs in similar ways [5].

Across sub-Saharan Africa (sSA), disruptions in health service delivery led to delayed care seeking, missed clinic visits, and reduced adherence to TB treatment [6–9]. The WHO report from 84 countries indicates that an estimated 1.4 million fewer people received care for tuberculosis (TB) in 2020 than in 2019 - a reduction of 21% from 2019 [10]. Similar disruptions in TB care were reported in Malawi where a 45.6% reduction in individuals presenting with presumptive pulmonary TB and a 19.1% decrease in patients registered for TB treatment was recorded during the COVID-19 pandemic [8]. In Zambia, a study investigating barriers to care during COVID-19 noted that TB patients were worried about contracting COVID-19 during clinic visits and frequently reported the impact of lockdowns on their financial security as a distressing change, with concerns regarding food scarcity and unstable housing for themselves and their families [11].

Uganda is a high TB burden country with a prevalence of 200 cases per 100,000 people [12]. In April 2020, shortly after the first confirmed COVID-19 case [13], the country implemented numerous measures to mitigate the spread of COVID-19. All public facilities e.g. schools, markets, and transport facilities were closed and all non-essential workers were asked to remain home for a long period or shelter at the workplaces for 3 months [9]. In addition, healthcare workers and equipment e.g. GeneXpert testing machines were reallocated to the COVID-19 emergency response [14]. These measures, while necessary, likely contributed to disruptions in TB care access leading to delays in TB diagnosis and linkage to care as well as reduced compliance with TB therapy [15]. A review of TB case notifications to the Uganda National TB and Leprosy Program(NTLP) reported a 43% reduction in TB case notifications in the six weeks following the institution of these mitigation measures [16]. While it is clear that these measures led to a reduction in TB case reporting there is very little research on the specific barriers that patients experienced. Our study aimed to understand patient-reported barriers to accessing TB care services during the COVID-19 pandemic in Uganda.

## Methods

### Study design

This was a cross-sectional mixed methods study employing both qualitative and quantitative data collection methods. A structured questionnaire was used to collect quantitative data and in-depth phone interviews (IDI) were conducted to identify patient-level barriers to access to TB care during the COVID-19 pandemic using the evidence-based COM-B theoretical framework, a comprehensive approach to behavior change.

### Study setting

This study was nested within a larger study describing the impact of COVID-19 mitigation measures on TB care seeking in Uganda being carried out at six TB treatment units in Uganda. Participants were enrolled from three public health facilities (two in the capital city Kampala and one rural health facility in Jinja district, eastern Uganda). These included two tertiary referral hospitals and one primary care facility. Each health facility has an outpatient clinic that offers TB diagnostic and treatment services free of charge. At these health facilities, the GeneXpert nucleic acid amplification testing algorithm is the diagnostic test used for all presumptive TB patients [17].

### Study participants

The study consecutively included TB patients who sought TB care from January 2020 to September 2020. Participants were classified as pre-lockdown (January- March 2020), lockdown (April-June 2020, and post-first lockdown (June-September 2020). In this analysis, we excluded participants without phone numbers, those who declined to have interviews, and without time of diagnosis. In the quantitative study, the inclusion criteria included adults 18 years and above, who received TB care at the study sites. In the qualitative study, we purposively selected participants who were either diagnosed with TB, missed a TB drug refill appointment, were admitted to an inpatient department, and visited a TB clinic during the COVID-19 pandemic. Participants were selected based on the period of TB care in relation to lockdown and health center location.

### Data collection

#### Quantitative data collection

We conducted phone interviews using a structured questionnaire that included questions addressing barriers to TB care.

#### Qualitative data collection

We purposively selected TB patients who had received TB care during the COVID-19 pandemic from (January 2020 to September 2020) and invited them to take part in in-depth interviews at three health facilities in Kampala and Jinja Districts. Participants were interviewed at the health facilities by one male and one female researcher (MB and SZM) with training and experience in qualitative research and fluency in both Luganda (the predominant local language) and English. Interviews were held in English, or Luganda according to the patients’ preference. Interviews were guided by an in-depth interview guide (supplementary file) that had previously been piloted in another health facility and included questions regarding barriers to TB care during the COVID-19 pandemic in Uganda (Supplementary Table 1). Each interview lasted about 30–40 min and was digitally recorded. We collected interviews until saturation was achieved.

### Data analysis

Quantitative data were analysed using STATA version 17.0 ^(R)^. For the categorical variables, we compared the association between the different categorical with the different periods using the chi-square test. Upon completion of interviews, audio recordings were transcribed and translated by study staff with language proficiency in the local language and English. A random audio sample was also compared with transcripts by study team member BM to ensure they corresponded with interview recordings. Three members of the research team (BM, NR, and RP) independently analyzed the transcripts using an inductive approach for content analysis. We resolved discrepancies through structured meetings to reach a mutual agreement and produce a codebook and identify themes related to patients’ experiences while receiving TB care.

## Results

### Quantitative results

710 participants were successfully contacted through phone calls and consented to participate in the study, and 672 completed the interviews and were thus included in this analysis. The median age of participants was 33 years (IQR: 26–42). The majority of participants were male (59.4%: 399/672), 70.8% (469/672) resided in urban areas and the median distance to the TB care facility was 5.6 (IQR: 3.0–11) kilometers (Tables 1 and 2). Pre-lockdown, lockdown, and post-first lockdown had similar baseline socio-demographic factors including distance to the health center, gender, HIV status, employment status, and socioeconomic status (SES) (*p* > 0.09) (Table 2).

**Table 1:** Demographic characteristics of study participants

Variable	n (%)
Age(years)	
mean (SD)	35 ± 11
Sex/gender	
Female Male	284(40) 419(60)
Residence	
Rural Urban	196(28) 507(72)
Employment status(n = 548)	
Unemployed Employed	151(28) 397(72)
Distance to the treatment center (Km)	
Mean (SD)	11 ± 23

**Table 2:** Socio-demographic characteristics of the participants by lockdown period

Variables	Pre-lockdown	lockdown	Post-first lockdown	Overall	*P*-values
**Age in years, median (IQR)**	32 (25–42)	33 (27–40)	34.5 (25–42)	33 (26–42)	0.090
**Distance from Health Care Facility in KM, median (IQR)**	6 (3–11)	5 (2.7–10)	5.2 (3.0–11)	5.6 (3.0–11)	0.475
**Area of residence**					
Rural	75(35.2)	63(33.3)	58(21.5)	196(29.2)	0.001
Urban	138(64.8)	126(66.7)	212(78.5)	467(70.8)	
**Gender**					
Female	91(42.7)	74(39.2)	108(40.0)	273(40.6)	0.740
Male	122(57.3)	115(60.85)	162(60.0)	399(59.4)	
**HIV status**					
Negative	125(67.6)	95(62.5)	118(63.1)	338(64.5)	0.553
Positive	60(32.4)	57(37.5)	69(36.9)	186(35.5)	
**Employment Status**					
Unemployed	46(42.9)	40(26.3)	61(32.6)	147(28.1)	0.213
Employed	139(75.1)	112(73.7)	126(67.4)	377(71.9)	
**Quantiles of SES**					
Lower	71(39.4)	43(29.1)	50(28.7)	164(32.7)	0.153
Middle	52(28.9)	55(37.2)	58(33.3)	165(32.9)	
Upper	57(31.7)	50(33.7)	66(38.0)	173(34.4)	

A majority of participants experienced one or more barriers to TB care 73.8% (496/672) as shown in Table 3. Table 3 further shows that the proportion of participants experiencing one or more barriers to TB care was 79.9% (151/189) during the lockdown which was significantly higher than pre-lockdown 68.1% (145/213) and post-lockdown 74.1% (200/270, *p* = 0.03). The most common barriers elicited included difficulties in accessing transport to the TB treatment clinics, the distance to the TB treatment units from their residences, and other reasons which are better explored in the qualitative results.

**Table 3:** Comparison of barriers to TB care during the COVID-19 lockdown and before the COVID-19 lockdown

Variables	Pre-lockdown n = 213	Lockdown n = 189	Post-lockdown n = 270	Overall n = 672	*P*-values
**Barriers Composite (1 or more)**	145 (68.1)	151 (79.9)	200 (74.1)	496 (73.8)	**0.027**
**Specific barrier**					
Transport	64 (30.1)	65 (34.4)	58 (21.5)	187 (27.8)	**0.007**
Distance to the health care facility	50 (23.5)	58 (30.7)	47 (17.4)	155 (23.1)	**0.004**
Costs of seeing a Doctor	1 (0.5)	3 (1.6)	2 (0.7)	6(0.8)	0.465
Lack Hospitals	0 (0.0)	0 (0.0)	3 (1.1)	3 (0.5)	0.106
Busy work	15 (7.0)	12 (6.4)	17 (6.3)	44 (6.6)	0.939
Physical Weakness	12 (5.6)	9 (4.8)	19 (7.0)	40 (6.0)	0.581
No family support	0 (0.0)	1 (0.5)	4 (1.5)	5 (0.7)	0.157
Not sure if doc help	1 (0.5)	3 (1.6)	1 (0.4)	5 (0.7)	0.280
Concern admission	1 (0.5)	2 (1.1)	1 (0.4)	4 (0.6)	0.615
Worry getting diseases	1 (0.5)	2 (1.1)	0 (0.0)	3 (0.5)	0.246
Try other medicine	29 (13.6)	25 (13.2)	31 (11.5)	85 (12.7)	0.752
No reason	8 (3.8)	6 (3.2)	10 (3.7)	24 (3.6)	0.941
Other	52 (24.4)	69 (36.5)	106 (39.3)	277 (33.8)	**0.002**

### Qualitative results

We conducted individual in-depth interviews with 28 participants, the majority 54% (n = 15) were male. The mean age of those undergoing interviews was 36 years (SD = 9). Most patients 78% (n = 22) received care during the lockdown (April-June 2020), 14% (n = 4) received TB care after the lockdown and 7% (n = 2) received TB care before the lockdown (before June 2020).

### Overview of the qualitative results

Participants reported transportation to the health center, cost of living, fear of COVID-19 transmission, the stigma surrounding respiratory symptoms, and accessibility of healthcare workers as significant barriers to TB care during the COVID-19 lockdown. Barriers to TB care during the COVID-19 lockdown are reported with supporting quotes below and summarized in the supplementary table.

### Barriers to TB care during the COVID-19 pandemic

#### Transportation to health center

Participants reported roadblocks, the threat of imprisonment or violence by law enforcement, and the cost of transportation as barriers to TB care access. Participants expressed increasing difficulty securing public or private transportation such as buses (matatus), taxis, and motorcycles (boda-bodas) during the COVID-19 lockdown.

*“[Before lockdown] It was easy a bit. But immediately I tried to join the hospital to start that medication from that health facility, that is when the lockdown came in…it was terrible. They could not allow boda-bodas to carry passengers, there were no taxis allowed. You had to explain too many words to those people who take control on the roads.” -Male, 37 years old, Admitted April-June*.

Specifically, individuals reported that roadblocks on main roadways and threats of imprisonment from law enforcement made transportation a major barrier to TB diagnosis and treatment. Some participants reported personally encountering the police which delayed or in some instances prevented them from attending TB clinic appointments. Others reported deferring travel due to the experiences of others in their community. These participants were included in the frequency calculations as they were deterred from travelling due to the possibility of encountering law enforcement.

*“We had to move at risk of being caught—we took shortcuts, being cautious of any motorcyclist that would ride by—the beating by the police officers in that area was very common and they did so with no consideration of any explanation even if the passenger was a patient. We would occasionally walk and arrive at a less risky lane….” -Male, 34 years old, Admitted*.

Participants reported certain factors were protective from disruption by roadblocks such as appearing more obviously ill.

*“I used the boda-boda < motorcycle > because by then the transport was locked; they were not allowing anyone to ride someone. However, there was some exception that the sick people they can be taken to hospital when they have the document to show that they are sick…whenever I could meet those police officers on the way, I could show them that I’m sick.” -Male, 23 years old, Diagnosed April-June*.

We found that women expressed fewer barriers related to transportation and reported fewer instances of being stopped at roadblocks compared to their male counterparts. Female participants cited a different set of barriers which included busy work, no family support, transportation, and economic insecurity as the most significant barriers to TB care access. A subset of participants felt that transportation barriers they experienced resulted in decreased TB medication adherence during the COVID-19 lockdown.

*“During that period is when the lockdown was enforced. In fact, I was not able to return. I tried to but I could not access any means of transport. There were neither motorcycles nor cars at the stage. So, I called that doctor of mine and told him that, ‘Doctor, I am challenged!’ I was told to return to the hospital on Monday and I have tried to but failed to access any means of transport to get there.” -Female, 47 years old, Admitted April-June*.

Additionally, participants report that transportation costs substantially increased during the COVID-19 lockdown compared to before the lockdown period. One participant described:

*“The boda-boda; it was costly because from Kabuusu to this place was Ugshs.7000(2USD) yet it was initially UgShs2000(0.6USD). But recall that you have a fever, you cannot walk but you have no option.” -Female, 27 years old, Diagnosed April-June*.

Due to the increasing cost of transportation during COVID-19 lockdown, most participants opted to walk long distances to health centres for TB treatment. As a result of these long walks, individuals described worsening TB symptoms, breathlessness, and exhaustion upon arrival at the health centre.

#### Financial constraints

During the COVID-19 lockdown the cost of food, and TB diagnosis were substantial barriers to participants and was compounded by increased costs of transportation to the health center. A few participants cited the high cost of TB diagnostic testing as a contributor to delays in TB diagnosis since it required solicitation of contributions from family and friends to pay for the TB diagnostic tests.

*“Money was scarce during that time. He did the biopsy test at Ugshs 160, 000(45USD), and after a week I received my results; the results indicated that I had TB but it had penetrated this organ (Testicular TB). Then I started treatment.” -Male, 52 years old, Missed July-Sept*.

Many participants described an increase in costs of living during the lockdown, specifically food and rent. For some participants, this contributed to decisions to relocate to rural settings, further away from TB treatment.

*“I could not afford to raise money for food and rent. So, the situation became too tough for me to handle.” -Male, 26 years old, Missed April-June*.

Many patients described an inability to afford healthy diets including fruits and vegetables which participants believed led to more side effects from TB medications prompting missed doses and in some cases cessation of TB treatment. Recommendations call for TB medications to be taken with food and the cost of food, therefore, presented another barrier to TB treatment and adherence. At TB treatment induction one participant described:

*“I was given a big tablet and instructed to swallow it by around 5 am to 6 am before eating anything. So, that became challenging for me hard for me since I was hungry and I had not eaten anything. That medication was always like my breakfast.” -Male, 26 years old, Missed April-June*.

Participants also described the dual burden of increasing costs of living with lost income due to unemployment during the COVID-19 lockdown. One participant stated:

*“The truth is that COVID hampered my proper treatment because we were not working yet that had been the source of financial support to facilitate my transportation to get medication. We found it very challenging. In fact, one time I called the doctor and told him about my challenge.” -Female, 47 years old, Admitted April-June*.

#### Fear of COVID-19 infection

Participants expressed fear of COVID-19 transmission during TB care. As a result, participants missed appointments to pick up TB medications. Participants described experiences of crowded waiting rooms at health centres and measures to mitigate COVID-19 exposure such as wearing masks and social distancing. Many participants reported their fear of COVID-19 was heightened given their current TB status. In response to travelling to the health centre, one participant said:

*“At that time, I could even fear to go to hospital to get medication because I feared that in case that sickness is topped with COVID—because I had two different kinds of illnesses—I had TB and diabetes. So, I had to safe guard myself. I was so vulnerable that I could easily contract another disease such as COVID.” -Male, 31 years old, Diagnosed April-June*.

Another participant also echoed the fear of COVID-19 transmission superimposed on TB:

*“Even when I had the transport, I was not motivated to go and then thirdly; there was also a threat of the dangerous COVID. I would think to myself that, ‘I may go with this disease and contract another disease.’’-Male, 34 years old, Missed April-June*.

#### The stigma of respiratory symptoms

Within the community, there were beliefs that COVID-19 transmission at health centers was common leading to reluctance to engage with health facilities. Some participants described confusion between symptoms of COVID-19 and other respiratory diseases leading to TB patients being quarantined at the health facilities.

*“I also feared that and expected it [diagnosis with COVID] because there was a popular rumour that ‘whenever you would go to the hospital even with just a mild cough, they would retain you at the hospital presuming that you were sick of COVID.’” - Female, 37 years old, Diagnosed April- June*.

Due to the similarity of respiratory symptoms among infectious and chronic respiratory diseases, participants with TB reported stigmatization. Individuals’ stigma related to active or residual TB respiratory symptoms being labelled as COVID-19 infection.

*“I used to fear a lot—I worried about contracting COVID…They would tell us to stay home safe and whenever I would hear people talk about it, I would even feel deeply hurt because whenever a person would hear you cough, they would assume that you were infected with COVID…I would worry so much about it.” -Male, 34 years old, Missed April-June*.

## Discussion

This mixed methods study explored patient-level barriers to accessing TB care services during the COVID-19 pandemic lockdown in Uganda. Quantitative data and qualitative data were collected to highlight factors limiting TB care and explore patient-level experiences during this period.

The quantitative results demonstrated that the proportion of individuals experiencing at least one barrier to TB care during the COVID-19 lockdown was significantly increased from 68.1% before the lockdown to 74.1% during the lockdown indicating a deleterious effect of COVID-19 lockdowns on care access. We found that access to and cost of transportation to the health facilities were commonly cited barriers, however, questionnaires revealed a high rate of “other barriers.” Our results remained similar to a retrospective study in Ethiopia found that compared to the pre-COVID time period TB detection, treatment success rate, and community health worker engagement in TB detection decreased by 11%, 17%, 77.2% [18]. Likewise, a mixed-methods study in our setting of Kampala, Uganda found that barriers to TB care included transport restrictions, transport cost, side effects of medications, unemployment and resulting poverty [19]. However, this study included a sample of refugees, which may have a greater number of barriers than our sample of community members diagnosed with TB.

Our results differed from work conducted within Uganda among HIV patients that showed the lockdown did not significantly limit HIV care in Uganda with viral load suppression improving by 68% during lockdown [20]. The reasons for this are not clear. This may be due to the adoption of the differentiated service delivery model under HIV programs in Uganda before and during COVID-19 which found people already receiving care in the community, unlike TB care which was and remains centralized. This may have facilitated extensions of HIV and TB care services to HIV/TB patients’ homes through the community linkage facilitators and village health teams [21]. This model is not yet fully adopted by the TB programs in Uganda. These types of interventions might significantly improve TB care delivery and post-implementation research will be important to determine their efficacy. These results also differ from work in Zambia that concluded an insignificant change in care seeking for TB patients during the COVID-19 pandemic [11]. The barriers to TB care during the lockdown described in this study such as limited transport to the facilities, distance to the facilities, costs to see the doctor and work obligations are similar to a study done in Ethiopia among TB patients seeking TB care services at TB treatment centers [22].

Our qualitative results describe significant barriers to care reported by TB patients during the COVID-19 lockdown. These include barriers that aligned with our phone questionnaire data such as transportation challenges, and costs of medications but also described new barriers such as increasing cost of living, fear of COVID-19 infection at health centers, the stigma of respiratory symptoms, and decreased accessibility of healthcare workers. This builds on the findings from prior qualitative studies in Madagascar and Brazil that identified barriers to TB care during the COVID-19 pandemic as stigma, treatment costs such as transportation and medications, food insecurity [23, 24].

Our findings suggest that patients experienced transport disruptions to the TB treatment center as the main barrier to TB care during the COVID-19 lockdown. This was due to increased cost of transportation, unavailable transport means, security roadblocks and the fear of security enforcement or imprisonment. Our results are in agreement with previous studies done in Uganda [19] and elsewhere [25–27], that COVID-19 lockdowns led to significant limitations on patients’ access to transportation to healthcare facilities.

COVID-19 lockdown affected many income generating activities, leading to financial challenges in the community. Simultaneously, due to supply chain and production challenges, the cost of living increased [28, 29]. Our qualitative study results show that patients experienced financial constraints during the pandemic which led to challenges paying for food, rent and other necessities. This limited their ability to visit TB clinics as well as appropriately adhere to medication.

The fear of COVID-19 infection was one of the major barriers to TB care identified during interviews. Several participants cited the fear of contracting COVID-19 at the health center and concerns regarding an increased susceptibility to COVID-19 due to TB infection. In addition, participants reported fear of being detained and quarantined if diagnosed with COVID-19. Our results are similar to work in Ethiopia among TB patients which identified fear of COVID-19 infection at health facilities as a strong predictor of missing TB medication appointments [22]. This finding is of particular concern given that delays in diagnosis of TB and avoidance of COVID-19 diagnosis lead to increased morbidity as well as household and community spread of both conditions.

The study had its strength and limitations. One strength is the use of mixed methods to collect questionnaire data from a large sample of patients from six urban and rural TB clinics throughout Uganda and to better describe barriers to care qualitative data from in-depth interviews was collected. Also, our use of in-depth interviews and qualitative analysis allowed for an improved understanding of other barriers by facilitating open-ended discussion as opposed to questionnaire-based data collection. This technique provides a more comprehensive understanding of the patient-level barriers to TB care during the COVID-19 pandemic. We utilized well-established statistical methods and rigorous qualitative methodology based on the theoretical framework and thematic analysis; a widely accepted systematic inductive method for analyzing qualitative data [30]. Despite these strengths, this study has some limitations. One limitation is during data collection IDIs were translated from Luganda to English from audio-recording of interviews. During the translation process, it is possible that meaning and specific local terminology may have been lost in translation during transcription. This was mitigated with a research team member speaking both Luganda and English reviewing transcripts. Our sample selection method was based on phone interviews which leads to selection bias and potentially under-represents subjects with lower socioeconomic status who do not have telephone access. We mitigated this by contacting the subject’s family members whose contact information was in the chart. The decision to use telephone-based recruitment was based on the risk of contracting COVID-19 and the high costs of personal protective equipment (PPE) during the study period. Still, future work to engage those without phones through community outreach is likely warranted. Our response rate was approximately 60% for those with phone numbers, this is a limitation, however, this is well in line with other phone-based studies and while potentially affecting results is unlikely to be mitigated by more aggressive recruitment [31].

## Conclusion

The COVID-19 pandemic significantly limited TB patients’ access to TB care services at TB treatment units in Uganda. The effect was exacerbated by the lockdown instituted to mitigate the transmission of COVID1-19 affecting mainly diagnosis, clinical appointments for scheduled medication refills and admissions due to TB-related illness. Transport access limitations, high cost of living, and fear of testing positive or getting infected with COVID-19 were strong barriers to seeking TB care. These results support the need for more additional approaches by stakeholders to ensure reliable access to healthcare for endemic infectious diseases even during the response to pandemics as well as interventions to tackle risk perception in the population affected emerging and reemerging infectious induced lockdown. The risk of further infectious disease-related lockdowns during emergency responses, further highlight the importance of investment into robust healthcare delivery systems and a comprehensive TB care model within the current HIV care delivery to combat barriers to TB care. Future studies should evaluate the effect of addressing these barriers to TB care during infectious disease-related lockdowns and impact on TB care uptake and participation.

### Electronic supplementary material

Below is the link to the electronic supplementary material.

Supplementary Material 1

Supplementary Material 2

## Data Availability

The datasets used and/or analyzed during the current study are available from the corresponding author on reasonable request.

## Declarations

### Patient and public involvement

Our research question and outcomes were developed through discussion with clinicians in the participating TB clinics and community health workers. Patients experience of the lockdown and barriers to TB care associated with this and other COVID19 mitigation measures was central to our research question and study plan. Patients were not involved in the design or recruitment of patients into this study, however, community healthcare workers did participate in our research plan and recruitment of subjects who underwent in depth interviews.
