# Supplementary material for: Patient level barriers to accessing TB care services during the COVID-19 pandemic in Uganda, a mixed methods study
Source: BMC Health Serv Res. 2024 Jan 10;24:52. doi: 10.1186/s12913-023-10513-8 (PMC10782633; doi:10.1186/s12913-023-10513-8)
Supplement: Supplementary file 1 — Supplementary Material 1 [file 12913_2023_10513_MOESM1_ESM.docx]

**Patient level barriers to accessing TB care services during the COVID-19 pandemic in Uganda, a mixed methods study: Mudarshiru B, Stella Z.M, et al.**

**Supplementary table 1.** Major themes of barriers to TB care during the COVID-19 lockdown in Uganda.

| **Major Themes, Subthemes and supporting quotes** | **Frequency of Theme** |
| --- | --- |
| **Barriers** |  |
| **Transportation to Health Center** | N=23 |
| ***Cost of transportation***   - “It took a period of about a month, then they…first took me to a health facility “*name withheld*” for treatment, and it was during the tough times of the first COVID-19 lockdown. Therefore, I was given a referral letter to a nearer health facility where I could receive treatment, however, at a fee but I did not have any money. Getting the transport fare was difficult, my sister—my father used to cover all the bills! I was brought here, and they started giving me TB treatment.” -*Female, 36 years old, Diagnosed during lockdown* - “I live in Kansanga; health facility “*name withheld*” is quite far. When I started treatment, it was during the lockdown so I had to walk from home to health facility “*name withheld*” and back. That is—because there was no means of transport; boarding a motorcycle was very costly. At the beginning, the motorcycles were prohibited from transporting people. Therefore, I had to walk to the hospital.” -*Male, 52 years old, Missed TB clinic visit during partial lockdown* - Transport was expensive, going to health facilities “*names withheld*” to pick your medicine was difficult! Because people would use pickups and it wasn’t free of charge, one would say, "Give me shs.60,000 and I take you to the health facility to pick your medicine" few people could do it since they couldn’t afford to. Due to those reasons, some people died, those that survived were still severely affected.” -*Male, 45 years old, Visited TB clinic during lockdown* | N=17 |
| ***Roadblocks and unavailable transportation***   - “During that period is when the lockdown was enforced. In fact, I was not able to return. I tried to but I could not access any means of transport. There were neither motorcycles nor cars at the stage. So I called that doctor of mine and told him that, ‘Doctor, I am challenged!’ I was told to return to the hospital on Monday and I have tried to but failed to access any means of transport to get there.” -*Female, 47 years old, Admitted during lockdown* - “Sometimes if you use the Pope Paul route, the roadblocks were there. So we would get a boda-boda, if you are almost approaching the roadblock, you get off the boda-boda and walk past them, then find the boda-boda waiting for you ahead.” -*Female, 27 years old, Diagnosed during the lockdown* - “The challenge was going to the hospital yet it is far away; I had to move yet sometimes the cars were prohibited from operating, as well as the motorcycles—it really inconvenienced me…I do not have the strength to walk from home to health facility “names withheld”.” -*Male, 29 years old, Visited and Admitted during the lockdown* | N=23 |
| ***Fear of police enforcement or imprisonment***   - “Most patients were always challenged because there are times I would meet some stopped by the traffic officers on the way. Those officers would not allow people to move by motorcycles even after showing proof of treatment documents.” -*Male, 28 years old, Diagnosed during lockdown* | N=12 |
| **Financial constraints** | N=12 |
| ***Increasing cost of food, rent, and basic necessities***   - “As you may be aware, the [TB] medication requires one to have a job in order to support themselves by purchasing food that is essentially required to sustain themselves on medication. I was greatly challenged because I did not have the financial support since, as you may recall, we were not working. I had to budget for and economize the little money I had—I did not know when I that situation would end. That is what challenged me the most but I fought so hard to adhere to my medication.” -*Male, 34 years old, Missed TB clinic visit during lockdown* - “The other challenges still lie within the failure to properly look after oneself, yet we patients are always told to take good care of ourselves; ‘Drink plenty, eat healthy’. That medicine is strong. Ensure that you take your medicine in time but it was hard for us to keep up because we could not afford to buy the required food yet the time to take the medication was due. So it became so difficult for us”- *Female, 47 years old, Admitted on TB ward during lockdown* - “They [patients with TB] stay at home. Some lack money for food, getting the money for transport is another challenge. Most people had challenges because going to the health facility is a priority, however, at the expense of the children staying hungry; you all die of hunger. It is poverty.” - *Male, 52 years old, Missed TB Clinic visit during partial lockdown* | N=12 |
| **Fear of COVID-19 infection** | N=6 |
| ***Fear of contracting COVID-19 at health center***   - “I was so scared! I got scared and said, ‘Eh! The people are so many! What am I going to do?’ And I said, ‘God please help me so that I don’t get COVID.’” -*Female, 36 years old, Diagnosed during lockdown* - “Even when I had the transport, I was not motivated to go and then thirdly; there was also a threat of the dangerous COVID. I would think to myself that, ‘I may go with this disease and contract another disease.’ So, I would go praying for God’s protection and intervention.” -*Male, 34 years old, Missed during lockdown* - “We were really fearing! Because we were told that those people with TB, they are very close to that disease—they are so friendly to that disease. Because it is a respiratory disease, and I'm already having a respiratory disease so they are too, too friendly. So we were advised to be so, so careful.” -*Male, 37 years old, Admitted during lockdown* | N=6 |
| ***Increased susceptibility of COVID-19 due to TB status***   - “You cannot fail to worry. Fear is inevitable because we are living in society, there are many patients at the hospitals so the risk of getting any other disease. However, that is also difficult because you are a patient, but leaving with another disease. So that is risky; you might even die because COVID is dangerous.” -*Male, 52 years old, Missed TB clinic visit during partial lockdown* - “We were really fearing! Because we were told that those people with TB, they are very close to that disease—they are so friendly to that disease. Because it is a respiratory disease, and I'm already having a respiratory disease so they are too, too friendly. So we were advised to be so, so careful.” -*Male, 37 years old, Admitted during lockdown* | N=2 |
| ***Fear of quarantine***   - “I also feared that and expected it [diagnosis with COVID] because there was a popular rumor that ‘whenever you would go to the hospital even with just a mild cough, they would retain you at the hospital presuming that you were sick of COVID.’” - *Female, 37 years old, Diagnosed during lockdown* | N=1 |
| ***Health care workers feared exposure to COVID-19***   - “.... however, when they took the sputum test, they asked for UGX20,000 and they had actually chased me away thinking it was Corona. My sister pleaded with them. So, the doctor put us aside"-*Female, 31years old, diagnosed during lockdown* | N=1 |
| **Health systems challenges** | N=8 |
| ***Limited admission space at hospitals***   - “Yes, it took us to Naguru where we were told that the beds had all been occupied [During Corona?] During Corona. So we continued to another facility..” *Female, 37years old, admitted during lockdown* - “It was difficult, however eventually, they made it easy for me and I got admitted. They got me a hospital bed but it was difficult-The people were in large numbers, yet the hospital beds were few. Until they assisted me and got me a place.” *Male, 49years old, Admitted during lockdown* | N=2 |
| ***Delays in diagnosis of TB***   - “Yes, then I went for an X-ray test, and I waited for 3days; they told me that they would call me.” *Female, 27years, diagnosed during lockdown* | N=3 |
| ***Few healthcare workers available at TB clinic***   - “I would go early [to the health center] but they would not handle all my issues because they were few healthcare workers and they had to attend to the patients in shifts… but I would not get all the required services because I could clearly see that it was impossible. So, I had to accept the services that they could available.” -Male, 34 years old, Missed April-June | N=3 |
