# Supplementary material for: Patient level barriers to accessing TB care services during the COVID-19 pandemic in Uganda, a mixed methods study
Source: BMC Health Serv Res. 2024 Jan 10;24:52. doi: 10.1186/s12913-023-10513-8 (PMC10782633; doi:10.1186/s12913-023-10513-8)
Supplement: Supplementary file 2 — Supplementary Material 2 [file 12913_2023_10513_MOESM2_ESM.pdf]

TBCoV observational study   In-depth Interview guide

The effect of the COVID19 pandemic on TB care in Uganda: New Diagnosis Interview Form

MAKERERE UNIVERSITY
SCHOOL OF MEDICINE
APPROVED
VALID UNTIL
17 MAR 2022
RESEARCH & ETHICS COMMITTEE
P.O.BOX 7072, KAMPALA

Subject ID:____________________

Name: ____________________

Interviewer: ____________________

Date: ____________________

Time Period (Circle one):

Pre-COVID-19 (Jan-March)   During COVID-19 (March-June)   After COVID19-19 (July-September)

Instructions:

(1) choose a setting with little distraction; (2) explain the purpose of the interview; (3) address terms of confidentiality; (4) explain the format of the interview; (5) indicate how long the interview usually takes; (6) tell them how to get in touch with you later if they want to; (7) ask them if they have any questions before you both get started with the interview

- Please use these questions as an outline, You may use prompts to solicit more information if needed
- Ensure all recording equipment is turned on prior to beginning the interview.
- Ensure the subject has had a refreshment and is seated comfortably in a quiet room for the interview
- Please ensure you are sitting over 2 meters away and the subject and the interviewer are wearing face coverings prior to the interview.

**Questions:**	COM-B Domain
1. Tell me what about your experience with being diagnosed with TB	*Data gathering
2. Did you find it easy or difficult to get your diagnosis of TB	Capability
3. About how long did you feel sick before you went to the doctor. How long did it take you to get diagnosed with TB? (may ask how long they had a cough or had sweats or felt weak etc.)	*Data gathering
4. About how long after being asked to get tested for TB did it take for you to get the results of your test	*Data gathering
5. Was there anything that made you wait to go see the doctor when you felt sick or made you go more quickly?	Motivation
6. Did you believe the doctor would help to make you feel better?	Motivation
7. Did the doctor explain your tuberculosis to you when you found out you had TB?	*Data gathering
8. Were you worried about getting more sick or catching a new disease if you went to the clinic or lab?	*Motivation
9. How easy or difficult was it to get to the clinic or lab where you were tested for TB?	Capability

7) Was it easy to get to the TB clinic and get taken care of? (if not why? if it was easy what made it easy?)	Capability
8) Tell me about your journey to get to the clinic? How long did it usually take to get to the TB clinic, was it sometimes a longer journey than other times? If so when did it take longer?	Opportunity, Capability
9) At any time did you delay going to TB clinic? If so why?	*Data Gathering
10) Do you think COVID19 made it more difficult to get to TB clinic? If yes how?	Capability

The effect of the COVID19 pandemic on TB care in Uganda: TB Ward Admission Interview Form

MAKERERE UNIVERSITY
SCHOOL OF MEDICINE
APPROVED
VALID UNTIL
17 MAR 2022
RESEARCH & ETHICS COMMITTEE
P.O.BOX 7072: KAMPALA

Subject ID:________________________

Name: ____________________

Interviewer: ________________

Date: ____________________

Time Period (Circle one):

Pre-COVID-19 (Jan-March) During COVID-19lockdown (March-June) After COVID-19 lockdown (July-September)

Instructions: (1) choose a setting with little distraction; (2) explain the purpose of the interview; (3) address terms of confidentiality; (4) explain the format of the interview; (5) indicate how long the interview usually takes; (6) tell them how to get in touch with you later if they want to; (7) ask them if they have any questions before you both get started with the interview

- Please use these questions as an outline, ensure all recording equipment is turned on prior to beginning the interview. You may use prompts to solicit more information if needed
- Ensure the subject has had a refreshment.
- Please ensure you are sitting over 2 meters away and the subject and the interviewer are wearing face coverings prior to the interview.

Questions:	COM-B Domain
1. Tell me what about your experience going to TB ward? (specified time period)?	*Data Gathering
2. Did you go to the doctor quickly or did it take a while? If you did delay why?	Opportunity
3. Did you find it easy or difficult to get to TB ward, tell me what made it hard or easy?	Capability
4. How long were you feeling sick before you went to the TB ward,	*Data Gathering
5. Were there lots of places to go to get care for your breathing problem/TB	Opportunity
6. Do you believe the doctor and hospital helped to make you feel better?	Motivation
7. Tell me about the medications you took for tuberculosis, were they difficult to take?	Data Gathering
8. Do you believe the medications you got in the TB ward helped you?	Motivation
9. Were you worried about getting more sick or catching a new disease if you went to the TB ward?	Motivation
